# Brain tissue haemoglobin expression in saline-perfused vs non-perfused rodents

**DOI:** 10.1016/j.heliyon.2023.e23343

**Published:** 2023-12-07

**Authors:** Marion Walser, Lars Karlsson, Reza Motalleb, Jörgen Isgaard, H Georg Kuhn, N. David Åberg

**Affiliations:** a Department of Internal Medicine, Institute of Medicine, The Sahlgrenska Academy, University of Gothenburg, Gothenburg, Sweden; b Department of Clinical Chemistry, Region Västra Götaland, Sahlgrenska University Hospital, Gothenburg, Sweden; c Department of Clinical Neuroscience, Institute of Neuroscience and Physiology, The Sahlgrenska Academy at University of Gothenburg, Sweden; d The Queen Silvia Children's Hospital, Sahlgrenska University Hospital, Gothenburg, Sweden; e Department of Specialist Medicine, Region Västra Götaland, Sahlgrenska University Hospital, Gothenburg, Sweden; f Hunter Medical Research Institute, University of Newcastle, Newcastle, NSW, Australia; g Institute for Public Health, Charité – Universitätsmedizin Berlin, Germany; h Department of Acute Medicine and Geriatrics, Region Västra Götaland, Sahlgrenska University Hospital, Gothenburg, Sweden

**Keywords:** Brain, Rodent, Saline-perfused, Non-perfused, Haemoglobin beta, 5′-aminolevulinate synthase 2

## Abstract

Haemoglobin beta (*Hbb*) and delta-aminolevulinate synthase 2 (*Alas2*) messenger RNA (mRNA) is mainly found in immature red blood cells, reticulocytes, and not in mature erythrocytes. However, these are also expressed in other tissues such as brain cells, mostly neurons. Therefore, exact quantification of neural tissue homogenates may be confounded by remaining blood in the brain vasculature that may give falsely high values of *Hbb/Alas2* expression. To investigate and compare the contribution of local *Hbb/Alas2* expression, we investigated mRNA expression locally in the hippocampus and prefrontal cortex, in post-sacrifice saline-perfused and non-perfused mice and rats. Although there was a higher level of *Hbb/Alas2* transcripts in the non-perfused animals, there was a significant mRNA expression in perfused brains that could at most partially be explained by remaining blood. Finally, we suggest that saline-perfusion should be recommended for quantification of brain *Hbb/Alas2* transcripts in homogenates.

## 1 Introduction

In the process of red blood cell formation, the so-called erythropoiesis, reticulocytes develop and mature in the bone marrow and then circulate for about 24 h in the bloodstream before developing into mature red blood cells. Reticulocytes constitute about 2.2 ± 1.2 % of all circulating blood cells [1]. Although the cell nucleus is lost when reticulocytes enter the bloodstream and mature into erythrocytes [2], there is some remaining mRNA, albeit very specialized, with at least 48.5–70 % of the mRNA coding for haemoglobin beta (Hbb) or delta-aminolevulinate synthase 2 (Alas2) [1,3]. Alas2 is a mitochondrial enzyme, required for Hbb synthesis in the heme biosynthetic pathway [4].

Apart from functioning as an oxygen transporter in erythrocytes, haemoglobin is also expressed in non-erythroid cells and can act as a redox enzyme due to its ability to bind hydrogen peroxide [5]. In the brain, *Hbb* is robustly expressed in neurons [6] and to a lesser degree also by astrocytes and may protect against oxidative stress [7]. The expression of *Hbb* in the brain is highly upregulated, for example by social stress in rats [8], growth hormone (GH) or insulin-like growth factor 1 (IGF-1) treatment [[9], [10], [11], [12]].

Regarding the analysis of haemoglobin-related transcripts in brain tissue, the possible influence of mRNA from residual erythrocytes in the vasculature needs to be considered. It has been demonstrated that about 2.5 % of brain volume in non-perfused rodents constitutes the blood volume [13], and that about 98.8–99.7 % of the blood volume in the hippocampus and neocortex is removed by post-sacrifice transcardial perfusion [14]. The methodologic problem of red blood cell contamination has often been managed by saline-perfusion of animals before sample collection, but no data currently exists in the literature comparing levels of haemoglobin-related transcripts in saline-perfused compared to non-perfused brain tissue.

In order to **investigate** more **reliable and** standardized measurements of brain tissue *Hbb* and *Alas2* mRNA levels, we **wanted to compare** the RNA levels of the two transcripts *Hbb* and *Alas2* in brain tissue from saline-perfused and non-perfused mice and rats. **The hypothesis was that saline-perfusion should significantly reduce the confounding of remaining red blood cells with respect to their high expression of *Hbb* and *Alas2* transcripts when quantifying brain tissue homogenate expression of these transcripts. For comparison, the house-keeping gene GAPDH, and the non-erythrocyte gene hypoxia-inducible factor-1 alpha (*Hif1-alpha*) were analysed, whose quantification of expression should not presumably be affected by saline-perfusion.**

## 2 Method

Male C57BL/6J (Charles River, Germany) mice at 6 months of age were housed under standard conditions of temperature (24 °C) with 50 %–60 % relative humidity. A 12-h light/dark cycle was maintained with lights from 19:00 to 07:00 (reversed), and with *ad libitum* access to food and water. In addition to mouse tissue, saline-perfused hippocampus (RR-203P) and non-perfused hippocampus (RR-203) from rats at 4 months of age and blood from rats (RR-705) and mice (MR-705-C57) was obtained as ready-to-use total RNA (AMSBIO, USA). The RNA had been isolated by modified guanidine thiocyanate techniques and stored in RNA storage buffer.

Upon time for sacrifice, mice were deeply anesthetized with a peritoneal injection of 50 mg/kg sodium thiopental during the active phase of the animals. Blood was extracted by cardiac puncture using a 27-gauge needle and collected in an EDTA/heparin tube. Following blood collection, animals were left non-perfused or immediately transcardially perfused with a cold saline solution (0.9 % NaCl). The perfusion was performed at a rate of 10 mL/min for 2–3 min per animal, corresponding to 20–30 mL. Additionally, perfusion was continued until 30 s after the perfusion fluid was completely clear. Brain tissue from pre-frontal cortex and hippocampus were micro dissected. Saline-perfused mouse brain tissues were immediately treated with RNAlater™ according to manufacturer's instructions and subsequently stored at −20 °C. Non-perfused mouse brain tissues were immediately snap-frozen in liquid nitrogen and subsequently transferred to −80 °C.

Total RNA was extracted from prefrontal cortex and hippocampus essentially according to Chomczynski and Sacchi with minor modifications as described previously [15,16]. Optical density (OD) of 260/280 measurements via NanoDrop 1000 (Thermo Scientific, USA) was used for quantification. cDNA was prepared from 250 ng total RNA using the HighCapacity cDNA Reverse Transcription Kit (Applied Biosystems, USA). Quantitative real-time PCR (qPCR) was used to quantify the transcripts, using a QuantStudio™ 3 Real-Time PCR System (Applied Biosystems, USA) with predesigned, TaqMan Gene Expression Assays (Applied Biosystems, USA; Table 1), for further details see http://www.appliedbiosystems.com. For details of qPCR quantification, see previously published Supplementary information, [10].

**Table 1:** Gene names and abbreviations of commercially available probes. The probes are assay-on-demand mixes of primers and TaqMan MGB probes (FAM dye-labeled). Further details can be obtained at htt.comp://www.appliedbiosystems. Key references for the transcripts.

Species	Gene symbol	Fullname	Assay number	Reference(s)
mouse	Hbb-b1	hemoglobin, beta adult major chain	Mm01611268_g1	[7,11]
	Alas2	5′-aminolevulinate synthase 2	Mm00802083_m1	[4,11]
	Gapdh	glyceraldehyde 3-phosphate dehydrogenase	Mm99999915_g1	
rat	Hbb-b1	hemoglobin, beta adult major chain	Rn 00583657_g1	[7,11]
	Alas2	5′-aminolevulinate synthase 2	Rn01637175_m1	[4,11]
	Hif1a	hypoxia-inducible factor 1, alpha subunit	Rn01472831_m1	[27]
	Gapdh	glyceraldehyde 3-phosphate dehydrogenase	Rn 01462662_g1	

The quantification cycle (Cq) value is the PCR cycle number at which the sample's reaction curve intersects the threshold line. The amount of product is doubled with each cycle, [17]. In our qPCR analysis, 40 cycles were run and with a lower level of transcripts, a higher Cq value was obtained. Descriptive data are shown for the Cq values of transcripts, followed by standard Student's t-tests as shown in Table 2.

**Table 2:** Descriptive data for quantification of transcripts.

Species	Unit	Tissue	Cond.	Gapdh	p	Hbb	p	Alas2	p	Hif1a	p
mouse	Cq values, mean ± SEM										
		blood	NA	28.1 ± 0.09		13.4 ± 0.09		19.3 ± 0.09		NA	NA
		hipp	P.	21.2 ± 0.06		28.4 ± 0.23		32.8 ± 0.23		NA	NA
		hipp	Non-P.	21.6 ± 0.07		26.2 ± 0.44		31.0 ± 0.44		NA	NA
	Relative Cq difference between tissue or condition. (binary diff./linear diff. [fold])										
		hipp; P vs Non-P		0.35/1.27	*	−2.16/4.47	**	−1.78/3.43	**	NA	NA
		blood vs hipp P		−6.82/113	***	15.0/3300	***	13.5/11830	***	NA	NA
		blood vs hipp non-P		−6.47/88.7	***	12.9/7380	***	11.8/3440	***	NA	NA
	Remaining transcript after perfusion (%)										
		hipp; P vs Non-P (=100)		22.4		22.4		29.1		NA	NA
	Cq values, mean ± SEM										
		PFC	P.	21.4 ± 0.06		28.0 ± 0.21		32.4 ± 0.21		NA	NA
		PFC	Non-P.	21.8 ± 0.10		26.2 ± 0.16		30.9 ± 0.15		NA	NA
	Relative Cq difference between tissue or condition. (binary diff./linear diff. [fold])										
		PFC; P vs Non-P		0.31/1.24	*	−1.83/3.56	***	−1.55/2.93	***	NA	NA
		blood vs PFC P		−6.61/97.8	***	14.7/25890	***	13.2/9350	***	NA	NA
		blood vs PFC non-P		−6.30/78.8	***	12.8/7280	***	11.6/3190	***	NA	NA
	Remaining transcript after perfusion (%)										
		PFC; P vs Non-P (=100)		124		28.1		34.2			
rat	Cq values, mean ± SEM										
		blood	NA	23.4 ± 0.04		13.3 ± 0.05		20.8 ± 0.04		31.1 ± 0.10	
		hipp	P.	21.8 ± 0.08		28.4 ± 0.04		33.8 ± 0.15		28.4 ± 0.01	
		hipp	Non-P.	21.7 ± 0.02		25.3 ± 0.02		31.2 ± 0.08		28.5 ± 0.05	
	Relative Cq difference between tissue or condition. (binary diff./linear diff. [fold])										
		hippocampus; P vs Non-P		−0.12/1.09	ns	−3.09/8.51	***	−2.56/5.90	***	0.12/1.09	ns
		blood vs hipp P		−1.65/3.14	***	15.1/35900	***	13.1/8780	***	−2.69/6.45	***
		blood vs hipp non-P		−1.77/3.41	***	12.0/4210	***	10.5/1490	***	−2.57/5.94	***
	Remaining transcript after perfusion (%)										
		hipp; P vs Non-P (=100)		92.0		11.7		17.0		108	

Footnotes: Abbreviations: Hipp; hippocampus, PFC; prefrontal cortex, P; perfused, non-P; non-perfused, ns; not significant, NA; not applicable.

For GAPDH, HBB, ALAS2, HIF1 alpha the detected Cq values are the original Cq values. For all transcripts the Cq values are expressed as mean ± standard error of the mean (SEM).

With respect to relative differences (Diff.) between transcripts and conditions, these are given as binary difference with respect to Cq values and in the decimal system (fold difference).

The remaining transcript after perfusion is given as a percentage comparison between non-P and P.

*P*-values derived from Student's T-test are shown with asterisks, * for p < 0.05, ** for p < 0.01, and *** for p < 0.001.

For hipp and PFC in mouse, the SEM originates from n = 12 mice per group, n = 6 mice in non-P PFC and n = 3 mice in non-P hipp, while for blood from mouse/rat and hipp from rat the SEM is from one biological sample, repeatedly measured 2–3 times. *P*-values are derived from T-tests (equal variance, two-tailed).

## 3 Results

We investigated mRNA transcripts by using specific RNA primers (Table 1) in whole blood from mice and rats, as well as prefrontal cortex and hippocampus in mice, and hippocampus in rats. Quantifications for *Gapdh*, *Hbb*, *Alas2* and *Hif1a* total RNA levels are shown in Table 2. There were substantially more *Hbb/Alas2* transcripts in the non-perfused vs. **saline-**perfused brain tissues. However, the amount that was left in the perfused tissue was still relatively high and well within the assessable part of the PCR amplification curve. The blood mRNA expression of *Hbb* in rats was 2^12^ times (≈4000-fold) higher than in the hippocampus of non-perfused rats and 2^15^ (≈36000-fold) higher than in perfused hippocampi. As the blood **was purchased and not collected from the perfusion experiments,** the exact comparison of levels however needs cautious interpretation. **For non-perfusion comparison to saline-**perfusion experiments, the comparisons, however, derive from the same experiments. After perfusion, 11.7–22.4 % of the detected *Hbb* remains in the hippocampus, and for *Alas2*, 17.0–29.1 % remains in the hippocampus. If *Hbb* would not be expressed within the brain tissue at all, the expected remaining blood of 0.3–1.3 % after perfusion would give a quantification of mRNAs in brain tissues with similar numbers [14]. Similar figures of remaining *Hbb* and *Alas2* post-perfusion were found in the pre-frontal cortex. Furthermore, this difference of expression comparing perfused and non-perfused tissues was not seen for the transcripts *Gapdh* or *Hif1a* which are indeed found in blood but confined to “normal” levels in reticulocytes, **rather** similar to the levels in brain tissue.

## 4 Discussion

**In our experiments we have compared saline-perfused and non-perfused brain tissue from two different rodent species with respect to the transcripts *Hbb* and *Alas2*, expressed abundantly in erythrocytes** [18] **and in neurons of the brain** [7,[19], [20], [21], [22], [23], [24]]**. The goal with our experiments was to show that saline-perfusion effectively reduces red blood cell confounding of brain tissue quantification of these transcripts, which was indeed the case. As expected, the expression levels of *Gapdh* and *Hif1-alpha* were essentially the same after saline-perfusion. Also, the experiments show that the remaining *Hbb* and *Alas2* expression after saline-perfusion, must be confined to other brain cell types than erythrocytes. Therefore, saline-perfused homogenates could be used for quantification of *Hbb* and *Alas2* in experimental treatments or external or internal changes in demand, for example oxygen supply or ischemia. Also, the similar results from two different species, mouse, and rat, and from two brain regions strengthen generalisability. Our results from qPCR,** show that the values of the erythrocyte-related transcripts are well within the measurable range in both the **saline-**perfused and the non-perfused brain tissue. However, **the relation between saline-perfused and non-perfused levels also** means that for optimal quantification of *Hbb/Alas2* in the brain tissue, perfusion is highly recommended. When analysing homogenates of brain tissues, no matter how diligent the perfusion is, there is probably some contribution of remaining blood (0.3–1.3 %) in the brain, which may partly confound quantification of *Hbb* and *Alas2* expression, but not **non-specific erythrocyte transcripts. The interpretation of possible blood contamination has also been acknowledged before** [25]**, but not been investigated in our manner. If there was a genuinely erythrocyte-specific gene, not expressed anywhere else in the brain, such a gene could be used as a reference for the efficacy of saline-perfusion. However, three recent investigations on 47 unique transcripts have found no such gene** [1,3,18]**, see Supplementary file for details. In fact, the genes previously thought to be specific for red blood cells, are examples of the ones that this project investigates, Hbb and Alas2.**

When it comes to limitations, it would have been an advantage if the blood had originated from the same rodents as the tissue. Although the very large differences between blood, perfused and non-perfused brain tissues needed very few samples for statistical detection, it would also have been valuable to have had a few more animals for more exact determination of percentages of remaining red blood cell transcripts. Another limitation of the study is that other quantification techniques could have been used. For example, Western blot quantifications of protein homogenates would have been valuable as a comparison. However, these would probably have presented an even higher haemoglobin level stemming from remaining erythrocytes, considering the extremely high haemoglobin protein content of all erythrocytes [26], as compared to the *Hbb* mRNAs only expressed by reticulocytes. Other techniques of quantification would have been in situ hybridization and immunohistochemistry. Although these would certainly have distinguished cell-type specific expression of the reticulocyte and brain cell mRNA expression, these have been well characterized previously [7].

## 5 Conclusion

We conclude that to measure brain transcripts that are numerously abundant in red blood cells, especially reticulocytes, it is recommendable to saline-perfuse the brain before brain tissue quantification. However, the experiments comparing non-perfusion and saline-perfusion also confirm that the red blood cell transcripts *Hbb* and *Alas2* show a robust non-erythrocyte brain expression as found by homogenates of brain tissue.

## Funding

The authors are grateful for grants from the Stiftelsen Peter Erikssons Minnesfond (NDÅ), grants from the Swedish Government and the county councils, the ALF-agreement (NDÅ: ALFGBG-965762, ALFGBG-719761 and HGK: ALFGBG-726541), the Swedish stroke association (NDÅ), 10.13039/501100003744Stiftelsen Hjalmar Svenssons forskningsfond (NDÅ: HJSV20200229) and Swedish medical research council (HGK: VR-MH 2019-01637).

## Animal experiments

All experiments were approved by the Gothenburg ethical committee on animal research (#181–2015) and performed in accordance with relevant guidelines and regulations.

## Declaration of competing interest

The authors declare the following financial interests/personal relationships which may be considered as potential competing interests:N. David Aberg reports financial support was provided by Stiftelsen Peter Erikssons Minnesfond. N. David Aberg reports financial support was provided by Government and the county councils. N. David Aberg reports financial support was provided by the Swedish stroke association. N. David Aberg reports financial support was provided by 10.13039/501100003744Stiftelsen Hjalmar Svenssons forskningsfond. H Georg Kuhn reports financial support was provided by Swedish medical research council. H Georg Kuhn reports financial support was provided by Government and the county councils.
